# A Mind–Body Intervention to Improve Physical Activity for Patients with Chronic Hip-Related Pain: Protocol for a Mixed Methods Study

**DOI:** 10.3390/jpm14050499

**Published:** 2024-05-09

**Authors:** Kate N. Jochimsen, Kristin R. Archer, Robin A. Pollini, Robert A. Parker, Nomin Enkhtsetseg, Cale A. Jacobs, Ana Maria Vranceanu

**Affiliations:** 1 Center for Health Outcomes and Interdisciplinary Research (CHOIR), Department of Psychiatry, Massachusetts General Hospital, Boston, MA 02114, USA; 2 Department of Psychiatry, Harvard Medical School, Boston, MA 02115, USA; 3 Department of Orthopaedic Surgery, Vanderbilt University Medical Center, Nashville, TN 37232, USA; 4 Department of Behavioral Medicine & Psychiatry, West Virginia University, Morgantown, WV 26506, USA; 5 Department of Epidemiology & Biostatistics, West Virginia University, Morgantown, WV 26506, USA; 6 Biostatistics Center, Massachusetts General Hospital, Boston, MA 02114, USA; 7 Mass General Brigham Sports Medicine, Boston, MA 02115, USA; 8 Department of Orthopaedic Surgery, Harvard Medical School, Boston, MA 02115, USA

**Keywords:** psychological skills, coping, rehabilitation, physical therapy

## Abstract

Background: Pain and dysfunction persist for most patients following hip-related pain treatment. Additionally, individuals with hip-related pain are typically less physically active than individuals without hip pain, despite evidence that regular physical activity reduces chronic musculoskeletal pain. Poor psychological health is common in patients with hip-related pain and further reinforces low physical activity. Mind–body interventions can improve psychological health and activity levels but have yet to be integrated to provide comprehensive, psychologically informed care for patients with hip-related pain. Thus, we are using the NCCIH intervention development framework to develop Helping Improve PSychological Health (HIPS), a novel, multimodal mind–body intervention to improve physical activity for individuals with hip-related pain and poor psychological health. Methods: We will recruit physical therapists (N = 20) and patients with hip-related pain (N = 20) to participate in 60 min qualitative interviews (focus groups with therapists; one-on-one interviews with patients). Using these data, we will develop the initial HIPS intervention and provider training materials. One physical therapist will be trained to deliver the HIPS intervention to five participants in an open pilot trial. Participants will attend six 30 min HIPS intervention sessions. We will collect quantitative data on satisfaction, improvement, and physical activity, alongside qualitative exit interviews with participants and the physical therapist in order to refine the HIPS intervention and provider training materials. Results: This study has been approved by the MGB IRB. We aim to develop and test the initial feasibility of the HIPS intervention in an open pilot trial. The findings from this project will inform a subsequent feasibility RCT.

## 1. Introduction

Hip-related pain accounts for at least 60% of hip pain cases in young- to middle-aged adults and is a precursor to osteoarthritis [1,2]. Current treatment models for individuals with hip-related pain focus primarily on increasing hip strength and range of motion through physical rehabilitation or on correcting joint morphology through surgery [3,4,5,6]. Both non-operative and surgical management fall short of providing ubiquitous benefits [3,7,8,9,10]. A 2019 systematic review demonstrated that non-operative treatment, including physical therapy and intra-articular hip injections, provides only limited and short-term improvement in pain and function [7]. Unfortunately, most patients experience continued pain and functional impairments and thus progress to surgery [11,12]. While surgery may improve local joint mechanics and reduce symptoms for some patients, disrupted movement persists, and three in four patients report unacceptable function two years after surgery [8,13]. One reason for these poor outcomes is that current treatments follow an outdated biomedical model largely ignoring the impact of psychological factors, including self-efficacy (a patient’s confidence in their ability to cope with their pain and complete daily tasks), pain catastrophizing (worst-case thinking and rumination on pain) and kinesiophobia (fear of painful movement and activity), despite evidence showing their pivotal role in a successful recovery [14,15,16]. Transitioning care to a biopsychosocial model, inclusive of psychological factors, has the potential to improve treatment outcomes for patients with hip-related pain.

Individuals with hip-related pain are also less physically active than healthy individuals [17,18,19]. It is not uncommon for patients with persistent pain to reduce their activity to minimize symptoms even though there is strong evidence that regular physical activity can decrease chronic musculoskeletal pain [20,21,22]. Unfortunately, current treatments for hip-related pain do not increase physical activity [18,23]. Poor psychological response to pain is common in patients with hip-related pain [15] and is a significant barrier to engagement in physical activity [24,25,26]. Current biomedical rehabilitation treatment models for hip-related pain do not include effective strategies to improve psychological factors or physical activity.

**Figure 1:**
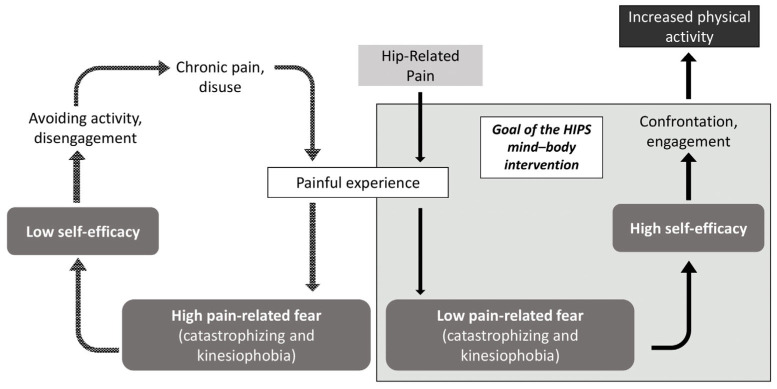
The goal of the HIPS mind–body intervention in the fear avoidance model.

**Figure 2:**
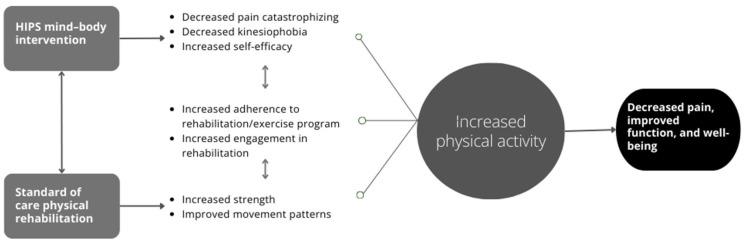
The conceptual model of the HIPS mind–body intervention.

Mind–body interventions improve psychological factors yet, to date, have not been implemented simultaneously with physical rehabilitation to provide comprehensive, psychologically informed care for patients with hip-related pain. The incorporation of mind–body interventions into psychologically informed practice has demonstrated preliminary efficacy [27]; however, evidence is lacking to support its broad adoption. Here we outline our plan to develop Helping Improve PSychological Health (HIPS), a mind–body intervention to improve physical activity for patients with chronic hip-related pain. HIPS will be delivered by physical therapists who are uniquely positioned to implement mind–body interventions because they meet frequently with patients and are trusted experts on recovery and rehabilitation. The HIPS intervention is designed specifically to improve psychological response to pain by targeting reductions in pain catastrophizing, reductions in kinesiophobia, and increases in pain self-efficacy. Based on the fear avoidance model (theoretical model; Figure 1) and our conceptual model (Figure 2), we anticipate that improvements in these mechanisms will result in improved physical activity, which, in turn, may improve pain and function.

Through the development of HIPS, we propose a paradigm shift in the care for patients with hip-related pain by providing a potentially effective solution to the current siloed, ineffective model of care, by harnessing the unique skill sets of physical therapists and capitalizing on the patient–provider relationship in physical rehabilitation. Below, we outline the first two steps of the HIPS mind–body intervention development, which include qualitative provider focus groups, patient interviews, and an open pilot trial with quantitative outcomes (satisfaction, overall improvement, and physical activity), as well as qualitative exit interviews (patients and provider) to test the initial feasibility of the HIPS intervention. The results will culminate in a future feasibility RCT.

### Theoretical Model and Treatment Targets

The HIPS mind–body intervention is grounded within the fear avoidance model (FAM) of chronic pain [28,29], which postulates that the psychological factors of self-efficacy, pain catastrophizing, and kinesiophobia perpetuate the cycle of avoidance behaviors, disuse, and disability (see Figure 1). These psychological factors are known barriers to successful recovery for individuals with chronic musculoskeletal pain, highlighting the fact that pathoanatomic features alone do not confer risk for poor treatment outcomes.

Following the FAM, the HIPS mind–body intervention will be multi-modal, integrating education and cognitive techniques (e.g., pain education and simplified cognitive restructuring), behavioral activation (e.g., SMART goal setting for physical activity and activity pacing), and relaxation/mindfulness techniques (e.g., progressive muscle relaxation, mindful awareness of pain)—components that individually improve self-efficacy, pain catastrophizing, or kinesiophobia (treatment targets/mechanisms of action). This multi-modal integration, and subsequent embedding of the HIPS mind–body intervention into routine physical rehabilitation, will synergistically improve physical activity (primary quantitative outcome) and enhance the standard of patient care (see Figure 2).

## 2. Materials and Methods

### 2.1. Inclusion and Exclusion Criteria

We plan to use the same patient eligibility criteria for both stages of the project (interviews and open pilot) described in this paper. We will recruit patients who have been diagnosed with a hip-related pain condition (femoroacetabular impingement syndrome, acetabular dysplasia, labral tear) from Mass General Brigham (MGB) and Massachusetts General Hospital (MGH) (Boston, MA, USA), as well as physicians and physical therapists from sports medicine and orthopaedic clinics in the greater Boston area. To be eligible, patients must be between 18 years old and older and have current hip pain (rated as a 30 out of 100 or higher on the Visual Analog Scale) that has lasted for a minimum of three months (chronic). They must also have poor psychological response to pain (Pain Catastrophizing Scale score ≥ 20 [30], Pain Self-Efficacy Scale score ≤ 40 [30], or 11-item Tampa Scale for Kinesiophobia score ≥ 17 [31]) and impaired physical activity (International Physical Activity Questionnaire Short Form (IPAQ-SF) score [32] < 150 min per week of moderate-to-vigorous physical activity [33], report that their hip pain interferes with their ability to be physically active, or be unsatisfied with their current level of physical activity). Exclusion criteria, which will be confirmed by the referring physician, physical therapist, or athletic trainer, include previous surgery on the symptomatic hip or having a non-intra-articular primary source of hip pain (e.g., lower back injury).

### 2.2. Recruitment and Screening

Patients: For both the interviews (N = 20) and open pilot trial (N = 5), patients will be recruited from their sports medicine and orthopaedic providers (physicians, physical therapists, athletic trainers) or research staff within the MGB and MGH sports medicine and orthopaedic clinics via study flyers. The patient study flyers for both the interviews and open pilot will include a QR code for interested patients to take the screening survey, housed in a REDCap database. A research assistant will also call patients who receive a study flyer to encourage them to complete the screening survey and answer any study-related questions they may have. Eligible and interested patients will be contacted by a research assistant and will provide informed consent. Anticipated Time Commitment: For the patient interviews, the total time commitment will be approximately one hour and 15 min (15 min for surveys/consent and 60 min interviews). For the open pilot trial, the total time commitment for participants will be approximately 5 h (six 30 min HIPS sessions, baseline and post-intervention surveys, and a 60 min interview).

Providers: For focus groups, physical therapists (N = 20; 4–5 groups) will be recruited by study flyers and word of mouth. For the open pilot trial, a physical therapist (N = 1) will be recruited from the MGB and MGH sports physical therapy clinics via word of mouth. Interested physical therapists who treat patients with hip-related pain will provide informed consent prior to participating in the focus groups or serving as an interventionist in the HIPS open pilot trial.

### 2.3. Study Designs

**Figure 3:**
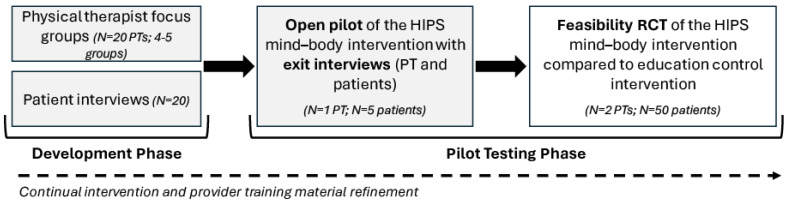
The phases of intervention development and pilot feasibility testing of the HIPS mind–body intervention; focus groups/interviews and open pilot trial with exit interviews (shaded in grey) are described in this paper.

In this project, we will develop and subsequently refine the HIPS mind–body intervention and provider training materials. These efforts will culminate in a future feasibility RCT of the HIPS mind–body intervention. Below is a description of each stage, and Figure 3 illustrates the phases of intervention development and testing.

(1) Intervention Development: We developed the conceptual model of the HIPS mind–body intervention (see Figure 2) using the established theoretical model (i.e., fear avoidance model; see Figure 1). Next, we identified potential psychological and mind–body skills to teach patients with chronic hip pain based on these conceptual and theoretical models. These are organized into three core evidence-based components: (1) behavioral activation, (2) education and cognitive techniques, and (3) mindfulness and relaxation techniques (see Section 2.4: HIPS Mind–body Intervention Components). Next, we will conduct 60 min qualitative interviews with patients (N = 20; one-on-one format). We will follow a semi-structured interview guide to gather information on a variety of topics including experiences with chronic hip pain treatment, HIPS mind–body intervention format preferences, perceptions of skills, and barriers and facilitators to physical activity (see Table 1). Using information from these interviews, we will develop the first version of the HIPS mind–body intervention manual.
(2) Training Development: Using evidence-based training models for psychologically informed practice [34,35], we developed a training framework that includes a three-day workshop co-led by the PI (Jochimsen) and clinical health psychologist (Vranceanu). We identified the broad educational concepts to be covered, including the theoretical and empirical background of the HIPS mind–body intervention, addressing potential challenges, and discussing model process factors (e.g., how to keep participants engaged, confidentiality, delivery of the active intervention) (see Section 2.5: Physical Therapist (Provider) Training). Next, we will conduct 60 min focus groups with physical therapists (N = 20; 3–4 focus groups). We will follow a semi-structured focus group guide to gather information on a variety of topics including facilitators and barriers to rehabilitation, experiences with mind–body interventions and psychologically informed practice, perceptions and comfortability with the skills, and training preferences. Using information from these focus groups, we will develop the first version of the HIPS provider training materials (provider training manual and fidelity checklist).
(3) Intervention and Training Refinement: We will use an open pilot trial with exit interviews and pre/post-assessments to optimize the HIPS mind–body intervention and refine the provider training materials. To facilitate the open pilot trial, we will train one physical therapist to deliver the HIPS mind–body intervention using the HIPS provider training materials. The physical therapist will be recruited via word of mouth from the MGB and MGH sports physical therapy clinics. Following provider training, we will enroll five patients with chronic hip-related pain. Patients will be recruited from their sports medicine and orthopaedic providers (physicians, physical therapists, athletic trainers) or research staff within the MGB and MGH sports medicine and orthopaedic clinics via study flyers. Following verbal consent, patients will be screened for eligibility using a screening survey housed in a REDCap database. Following screening, interested and eligible patients will provide written informed consent to participate in the open pilot study. They will complete baseline surveys (see Table 2) and receive a waist-worn activity monitor (ActiGraph xGT3X-BT) to wear on a waistband centered over their painful hip for 7 consecutive days, removing it only to sleep and shower. All six HIPS mind–body intervention sessions will be scheduled. Sessions will be delivered in a private room dedicated for research and will be scheduled at the convenience of the patient, ideally before or following their routine physical therapy appointment. To minimize variability in physical rehabilitation during the open pilot trial, physical therapists providing routine, concurrent physical therapy will be provided the recently updated Clinical Consensus Guidelines for treating non-arthritic hip conditions [36]. They will use these guidelines as a framework for their patient-specific treatment plan. Physical therapist notes will also be accessed to document treatment goals, exercise progressions, home exercise programs, and number of visits attended. We will track session dates/times to consider whether intervention timing (e.g., patient fatigue) plays a role in treatment feasibility, acceptability, or satisfaction. All intervention sessions will be audio-recorded, and the physical therapist will complete the fidelity checklist after each session. Following the last HIPS mind–body intervention session, participants will repeat baseline surveys, the Client Satisfaction Questionnaire, the Global Rating of Change Scale (to measure overall improvement), and a 7-day wear of the ActiGraph accelerometer. We will also conduct qualitative exit interviews with the physical therapist and patients to gauge clinician comfort in delivering the intervention and further refine the provider training protocol and manual, as well as further refine the HIPS mind–body intervention materials. Participant interview questions will focus on intervention delivery format, content usefulness and clarity, and barriers to meeting physical activity goals. We will meet with patients and physical therapists to discuss and review refinements of the HIPS intervention.
(4) Retention Strategies: To retain participants throughout the HIPS intervention, we will use methods of retention employed in other NIH-funded studies in orthopedics including (1) participant reimbursement for assessments, (2) a clinical research coordinator trained in communication strategies to build rapport with participants and increase retention, (3) reminder emails or texts for HIPS sessions and assessments, and (4) flexibility with scheduling session times. Missing data will be minimized using REDCap to electronically collect surveys (required fields). We will also use data collected in the open pilot exit interviews to examine barriers to attending HIPS sessions and achieving physical activity SMART goals. We will address these prior to the feasibility RCT.

### 2.4. HIPS Mind–Body Intervention Components

The HIPS mind–body intervention incorporates three core evidence-based components and will be optimized to address the specific needs and preferences of individuals with chronic hip-related pain and poor psychological response to pain. All components of the HIPS mind–body intervention fall in the scope of physical therapy. Psychological aspects of rehabilitation, including goal setting and the neurophysiology of pain, are included in physical therapy curriculums. However, physical therapists may have less experience and therefore require more intensive training in mindfulness, relaxation, and adaptive thinking techniques. This outline of the HIPS mind–body intervention will be refined following qualitative interviews and then again after the open pilot trial.

(1) Behavioral Activation (~10 min per session): Participants will be encouraged to use the SMART (Specific, Measurable, Actionable, Realistic, Time-bound) framework to set both performance and process goals to decrease sedentary time/increase light physical activity. These will be set and evaluated during each intervention session. Physical activity pacing will be used to progress the duration and frequency of physical activity.
(2) Education and Cognitive Techniques (~10 min per session): Pain education will be used to help patients re-conceptualize their pain experience, thereby decreasing the threat of pain, encouraging healthy movement, and facilitating focused engagement in rehabilitation. Participants will also be taught simplified cognitive restructuring. For example, patients will be taught to identify worst-case or fear-based thinking (e.g., pain means that I have injured my hip worse), challenging this thought based on their pain education, and replacing them with adaptive thoughts (e.g., pain does not equal tissue damage; my body is safe).
(3) Mindfulness and Relaxation Techniques (~10 min per session): Mindfulness practices including self-compassion, mindful awareness of pain, mindful walking, progressive relaxation, diaphragmatic breathing, and body scanning will be incorporated. Participants will learn general mindfulness and relaxation techniques, as well as when to use each for maximal benefit (e.g., use self-compassion when not meeting SMART goals, using diaphragmatic breathing when noticing anxiety about pain).

### 2.5. Physical Therapist (Provider) Training

In the open pilot trial, one physical therapist will be trained on the HIPS mind–body intervention using the provider training protocol, provider training fidelity checklist, and provider manual. This provider training will be co-led by the primary investigator (Jochimsen) and clinical psychologist (Vranceanu). Consistent with current evidence-based psychologically informed practice training models [34,35], the physical therapist for the open pilot trial will attend a three-day workshop where we will describe the theoretical and empirical background of the HIPS mind–body intervention, review the provider training manual content, review potential challenges and how they might be addressed, and discuss model process factors (e.g., how to keep participants engaged, confidentiality, delivery of the active intervention). Throughout the training, skills will be reinforced via experiential learning and role-playing how to teach and incorporate skills into rehabilitation. The physical therapist will also receive supplementary printed materials to reference. To assess fidelity of the provider training, the training session will be audio recorded and checked against the provider training fidelity checklist.

### 2.6. Power Analysis

While a power analysis is not indicated for qualitative studies, an N = 20 patients and N = 20 physical therapists (4–5 groups) will be sufficient to achieve saturation of pre-determined domains. The purpose of the open pilot trial (N = 5 patients) is to evaluate initial feasibility (e.g., Can we recruit? Is the intervention credible? Are patients satisfied?) and then use qualitative data to understand ways feasibility can be further increased and the intervention optimized. Thus, our open pilot exit interviews with 5 patients should be sufficient to achieve our goal of evaluating initial feasibility, especially given the homogenous study population (all receiving the HIPS intervention) recruited from a single geographic area [50]. These sample sizes are consistent with the stage of intervention development following the NIH Stage Model for Behavioral Intervention Development (Stage 1A: Intervention Generation/Refinement) [51]. These sample sizes are also consistent with other, similar NIH-funded intervention development trials [52,53]. We have established methods for participant retention (see Retention Strategies). However, should recruitment be slower than anticipated, we will expand recruitment to additional physical therapy and sports medicine/orthopedic clinics.

### 2.7. Data Analysis

#### 2.7.1. Qualitative Focus Groups and Interviews

We will transcribe and de-identify all transcripts which will then be uploaded to the Dedoose qualitative analysis software, version 9.0.107. We will subsequently use thematic analysis to code the data using a hybrid inductive/deductive approach based on the framework method [54]. Specifically, we will be deductive by using prior research and our theoretical model to inform our interview guide, rapid data analysis template, and codebook domains. The pre-determined domains for the physical therapist focus groups include (1) facilitators and barriers to rehabilitation, (2) experiences with psychologically informed practice, and (3) content and training preferences for psychological skills and mind–body interventions. The pre-determined domains for the patient qualitative interviews include (1) challenges and impact of living with chronic hip pain, (2) previous treatment experiences, and (3) preferences for psychological skills and mind–body interventions. These domains are aligned with the drafted questions included in Table 1. We will also allow for new themes to emerge during the interview process. Throughout the process, we will iteratively discuss and review the codebook and each transcript. To ensure the rigor of the qualitative data analysis, two team members will independently code the transcripts. Any discrepancies will be resolved through group discussion and comparison to raw data. The coded data will be refined into themes and subthemes within each of the predefined domains, which will be used to guide the development of the HIPS mind–body intervention and provider training materials. For example, if patients or physical therapists express that they would prefer to have the flexibility of virtual interventions or training, this may be added as an option.

#### 2.7.2. Open Pilot Trial

To determine the initial feasibility of the HIPS mind–body intervention, we will report the number of patients that were approached, screened, and eligible, as well as the number of participants enrolled. We will also report the number and percentage of participants who completed at least 4 of 6 intervention sessions. Participants who drop out will be counted as not meeting applicable feasibility criteria. Full feasibility, fidelity, and acceptability benchmarks are located in Table 2. In addition to these benchmarks, we will transcribe and de-identify the patient exit interview transcripts, which will then be uploaded to the Dedoose qualitative analysis software. These transcripts will be coded in the same process that Is described above. Pre-determined domains for patient exit interviews include (1) experiences with the HIPS mind–body intervention (delivery format and content usefulness and clarity), (2) barriers and facilitators to meeting physical activity goals, and (3) future treatment plans and expectations. To determine the initial feasibility and fidelity of the provider training, we will report adherence to the provider training fidelity checklist by comparing the checklist to the audio recording of the training session. A research assistant will also evaluate recorded HIPS mind–body intervention sessions and compare them with the intervention fidelity checklist throughout the open pilot trial. We will analyze the physical therapist qualitative exit interview in the same manner as described above, with a focus on specific pre-determined domains: (1) HIPS provider training experience, (2) the physical therapist’s level of comfort in delivering the HIPS mind–body intervention, and (3) challenges and barriers with delivering the HIPS mind–body intervention. For quantitative pre/post measures (see Table 2) we will run descriptive statistics and exploratory correlations to determine whether our conceptual model (see Figure 2) is supported by the data. Additionally, we will report the number of valid days (minimum 10 h of wear time) participants wore their ActiGraph accelerometers at baseline and post-intervention testing sessions, the average number of physical therapy visits attended, the proportion of participants that adhered to their prescribed home exercise program, and the proportion of participants that progress to surgical intervention. We will then use triangulation to merge patient qualitative (exit interviews) and quantitative (Client Satisfaction Questionnaire) data, and to merge provider qualitative (exit interview) and quantitative (intervention fidelity checklist) data. Triangulation will be used to integrate qualitative and quantitative data providing a robust, more comprehensive understanding of the initial feasibility, fidelity, and acceptability of the HIPS mind–body intervention to guide future refinements. To triangulate our qualitative and quantitative data, we will construct a matrix in which columns are labeled for key quantitative findings and rows are labeled for codes generated in the qualitative thematic analysis. We will note points of convergence (i.e., a cell of a matrix where both analysis types point to the existence of positive, negative, or no association) and divergence and conduct secondary analyses to formulate and explore hypotheses for any observed divergences.

### 2.8. Scientific Rigor

To ensure rigor and reproducibility, we will use valid and reliable patient-reported outcome tools stored in a secure REDCap database, use valid and objective physical activity measures (ActiGraph xGT3X-BT accelerometer), assess the reliability of qualitative interviews by using an independent coder, and use a manual and test fidelity for provider training and HIPS mind–body intervention delivery.

## 3. Results

We have begun interviews, which is the first step in the HIPS intervention development. The aim of this project is to establish the initial feasibility of the HIPS mind–body intervention in an open pilot trial. The target date of completion for the open pilot trial is December 2024. The findings from this project will inform the refined HIPS mind–body intervention and provider training materials, which will be tested in a subsequent feasibility RCT (N = 50 patients), which will be registered on clincialtrials.gov.

## 4. Discussion

Chronic hip pain impacts patients’ ability to be physically active, and current treatment options do not improve this. Prior research suggests that psychological factors play a pivotal role in patients’ treatment response, yet there are no feasible and scalable evidence-based interventions to address psychological factors and improve physical activity in patients with chronic hip-related pain. This paper describes a mixed methods study that will develop and test the initial feasibility of the HIPS mind–body intervention. The HIPS intervention aims to fill this gap and help patients with chronic hip-related pain regain their physical activity, ultimately reducing their pain and improving their well-being.

Potential Benefits for Participants: Though the goal of this project is to develop and optimize the HIPS mind–body intervention and the provider training materials, participants in the open pilot trial may benefit via improvements in their psychological response to pain (e.g., decreased pain catastrophizing, decreased kinesiophobia, and/or increased pain self-efficacy). By improving these treatment targets and using behavioral activation techniques in the HIPS mind–body intervention, we hypothesize that participants will increase their physical activity, potentially resulting in reduced hip pain and improved function (see conceptual model; Figure 2).

This study protocol will be valuable to future mind–body and psychological skills intervention development, especially within rehabilitation sciences. We have carefully described the study procedures, including recruitment, screening, data collection, and data analysis. In addition, we have provided an overview of the HIPS intervention components and detailed plans to iteratively refine the intervention.

The HIPS mind–body intervention takes a holistic, whole-person approach to treating chronic musculoskeletal pain, similar to other psychosocial interventions in patients with acute traumatic orthopedic injuries [55] and chronic lower back pain [56]. The HIPS mind–body intervention will be uniquely tailored to the needs and preferences of this specific patient population and the physical therapists treating them.

In summary, this mixed-methods study aims to gather data from clinicians and patients to methodically create a feasible intervention that is tailored to the specific needs of physical therapists (in terms of delivery) and patients with chronic hip-related pain (in terms of content). Following the completion of this project, it is our goal to progress to a single-site feasibility trial. Improving psychological factors for patients with chronic hip-related pain has a strong potential to increase physical activity and thereby reduce pain and improve physical function and well-being. The proposed HIPS mind–body intervention delivered by physical therapists may be an effective and scalable solution to improve physical activity through psychologically informed practice to promote patient well-being for the millions of patients with chronic hip pain.

## Figures and Tables

**Table 1:** Example questions.

Physical Therapist Qualitative Focus Group Questions
▪ What do you think prevents patients from responding well to rehabilitation for their chronic hip pain?
▪ How do you think your patient’s mood impacts their recovery?
▪ Do you use any mind–body or psychological skills interventions in your practice? [PROMPTS] If no, why not? If yes, which interventions do you use and how do patients respond?
▪ What are your impressions of the HIPS mind–body intervention?
▪ If you were trained on the HIPS mind–body intervention, what would your training preferences be?
▪ What barriers do you foresee in implementing the HIPS mind–body intervention, or adopting a psychologically informed practice?
**Patient qualitative interview questions**
▪ How does your chronic hip pain impact your life? [PROMPTS] Ability to be physically active, relationships, mood, quality of life.
▪ What barriers do you face when trying to be physically active?
▪ What treatments have you tried for your hip pain, and what were your expectations for these treatments?
▪ Have you ever tried any mind–body or psychological skills interventions? (examples provided)
▪ What are your impressions of the HIPS mind–body intervention?
▪ What intervention format would you prefer, and what format would be most convenient for your life?

**Table 2:** Outcome measures.

Construct	Measure Description
**Covariates**	
Demographics	Age, biological sex (because hip-related pain is more common in females) [37], gender, race/ethnicity, education level, employment status
Clinical variables	Symptom duration, clinical diagnosis, previous treatments, physical therapy attendance during the HIPS trial (# of sessions attended, home exercise program, progression of exercises), mental health history
**Primary Outcomes**	
Feasibility *≥70% excellent*	▪ Feasibility of recruitment: proportion of participants who agree to participate from the total number of patients approached ▪ Feasibility of data collection: proportion of participants who complete all surveys
Fidelity *≥90% excellent*	▪ Proportion of intervention sessions with 100% of the content delivered
Acceptability *≥80% excellent*	▪ Satisfaction: proportion of participants with Client Satisfaction Questionnaire [38] scores above the midpoint ▪ Retention: proportion of the participations who attend at least 4 of 6 sessions ▪ Improvement: proportion of participants who report overall improvement on the Global Rating of Change Scale [39]
**Secondary Outcomes**	
Physical activity	▪ ActiGraph accelerometry (ActiGraph xGT3X-BT) 7-day wear—objective measure of sedentary time, light physical activity, and moderate-to-vigorous physical activity [40] ▪ International Physical Activity Questionnaire Short Form (IPAQ-SF)—self-reported measure of physical activity (minutes of sedentary, light, and moderate-to-vigorous physical activity per week) [32] ▪ Pain interference with physical activity—self-reported “Does your hip pain interfere with your ability to be physically active?” (yes/no) ▪ Satisfaction with physical activity—self-reported “Are you satisfied with your current level of physical activity?” (yes/no)
Pain	▪ Hip pain measured as current, average over the last week, and worst on a 10-point Visual Analog Scale (0 indicating no pain, 10 indicating the worst pain imaginable) [41] ▪ Pain interference will be measured using the 6-item PROMIS Pain Inference tool [42] ▪ 33-item International Hip Outcome Tool (iHOT-33)—measures hip symptoms including hip pain, function, and quality of life [43]
Psychological factors *(mechanisms of action)*	▪ Pain Catastrophizing Scale (PCS)—measures worst-case thinking, inability to divert attention from pain. The PCS consists of 13 items rated on a scale from 0 (not at all) to 4 (all the time). Total scores range from 0 (low pain catastrophizing) to 52 (high pain catastrophizing) [44] ▪ Pain Self-Efficacy Questionnaire (PSEQ)—measures a patient’s confidence in their ability to cope with their pain and perform daily tasks despite their pain. The PSEQ consists of 10 items rated on a scale from 0 (not at all confident) to 6 (completely confident). Total scores range from 0 (low self-efficacy) to 60 (high self-efficacy) [45,46] ▪ 11-item Tampa Scale for Kinesiophobia (TSK-11)—measures fear of painful movement or activity. The TSK-11 has 11 items rated on a scale from 1 (strongly disagree) to 4 (strongly agree). Total scores range from 11 (low kinesiophobia) to 44 (high kinesiophobia) [47,48]
Well-being	▪ Patient-reported psychological well-being will be measured with the 5-item World Health Organization Well-Being Index (WHO-5). The WHO-5 has 5 items rated on a scale from all of the time (5) to at no time (0). Percentage scores are calculated and range from 0 (low well-being) to 100 (high well-being) [49]

## Data Availability

There are no data included in this article.
